# Flexible multifunctional titania nanotube array platform for biological interfacing

**DOI:** 10.1557/s43577-023-00628-y

**Published:** 2023-12-08

**Authors:** Hoda Amani Hamedani, Thomas Stegall, Yi Yang, Haochen Wang, Ashwin Menon, Anubhuti Bhalotia, Efstathios Karathanasis, Jeffrey R. Capadona, Allison Hess-Dunning

**Affiliations:** 1 Advanced Platform Technology Center, Louis Stokes Cleveland Veterans Affairs Medical Center, Cleveland, USA; 2 Department of Materials Science and Engineering, Case Western Reserve University, Cleveland, USA; 3 Department of Biomedical Engineering, Case Western Reserve University, Cleveland, USA; 4 Department of Electrical, Computer, and Systems Engineering, Case Western Reserve University, Cleveland, USA; 5 Department of Mechanical Engineering, Case Western Reserve University, Cleveland, USA; 6 Case Comprehensive Cancer Center, School of Medicine, Case Western Reserve University, Cleveland, USA

**Keywords:** Nanostructure, Electrochemical synthesis, Flexible device, Interface, Lithography

## Abstract

**Abstract:**

The current work presents a novel flexible multifunctional platform for biological interface applications. The use of titania nanotube arrays (TNAs) as a multifunctional material is explored for soft-tissue interface applications. *In vitro* biocompatibility of TNAs to brain-derived cells was first examined by culturing microglia cells—the resident immune cells of the central nervous system on the surface of TNAs. The release profile of an anti-inflammatory drug, dexamethasone from TNAs-on-polyimide substrates, was then evaluated under different bending modes. Flexible TNAs-on-polyimide sustained a linear release of anti-inflammatory dexamethasone up to ~11 days under different bending conditions. Finally, microfabrication processes for patterning and transferring TNA microsegments were developed to facilitate structural stability during device flexing and to expand the set of compatible polymer substrates. The techniques developed in this study can be applied to integrate TNAs or other similar nanoporous inorganic films onto various polymer substrates.

**Impact statement:**

Titania nanotube arrays (TNAs) are highly tunable and biocompatible structures that lend themselves to multifunctional implementation in implanted devices. A particularly important aspect of titania nanotubes is their ability to serve as nano-reservoirs for drugs or other therapeutic agents that slowly release after implantation. To date, TNAs have been used to promote integration with rigid, dense tissues for dental and orthopedic applications. This work aims to expand the implant applications that can benefit from TNAs by integrating them onto soft polymer substrates, thereby promoting compatibility with soft tissues. The successful direct growth and integration of TNAs on polymer substrates mark a critical step toward developing mechanically compliant implantable systems with drug delivery from nanostructured inorganic functional materials. Diffusion-driven release kinetics and the high drug-loading efficiency of TNAs offer tremendous potential for sustained drug delivery for scientific investigations, to treat injury and disease, and to promote device integration with biological tissues. This work opens new opportunities for developing novel and more effective implanted devices that can significantly improve patient outcomes and quality of life.

**Graphical abstract:**

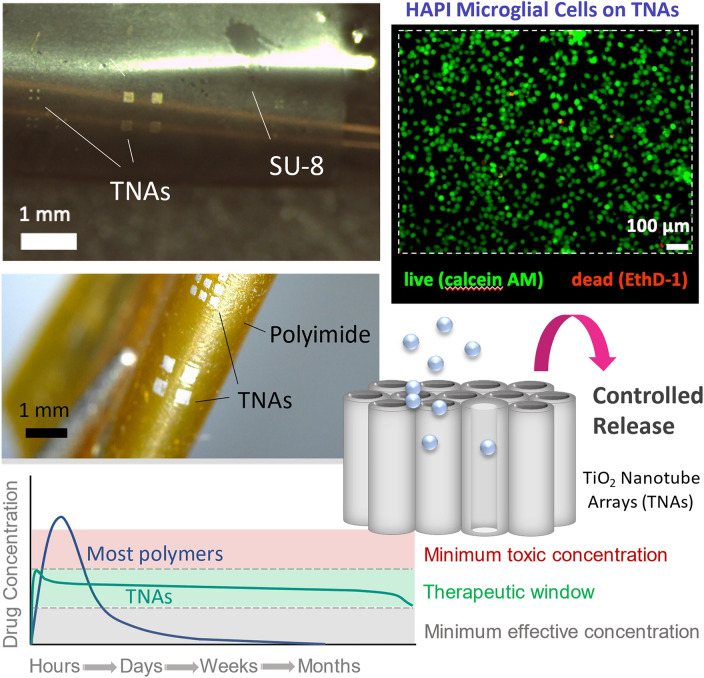

**Supplementary information:**

The online version contains supplementary material available at 10.1557/s43577-023-00628-y.

## Introduction

Titania (TiO_2_) has been widely used in pharmaceutical and medical applications.^1^ The utilization of titania has been extensively explored in biomaterial applications pertaining to hard tissues. Titania scaffolds and coatings are widely recognized for their ability to effectively interface between connective tissue and rigid structural replacement fixtures, including dental and orthopedic implants, as well as vascular stents.^2^ This is owing to the biocompatibility, anti-inflammatory, and antibacterial properties of the material, as well as the mechanical and thermal stability of titania structures. Additionally, titania nanostructures with nanoporous topography have been shown to promote bone cell adhesion, differentiation, and proliferation, as well as osseointegration and hemocompatibility.^3–5^ These properties are further enhanced in vertically aligned TiO_2_ nanotube arrays (TNAs), which are regarded as the most promising types of nanoporous inorganic coatings for controlled and sustained pharmacologic release from implants.^6^ TNAs are regarded as a high-capacity drug delivery vehicle compared to polymers.^7^ The high surface area with tunable surface chemistry and geometry of the nanotubes, such as pore size and tube length, allows for precise modulation of drug-release kinetics, thereby, delivery of therapeutic molecules with desired doses for various periods of time, from days to months.^8–10^ In applications where sustained release is required, TNAs offer an advantage over traditional hydrogel-based drug delivery platforms because of their higher loading efficiency and the ability to control release of small molecules (or approximating ligands as spheres of hydrodynamic radius <1 nm), which constitute the majority of pharmaceutical drugs.^11–13^ Unlike drug-eluting hydrogels, TNAs are not dependent on swelling to release their payloads; therefore, problems such as low mechanical strength in the swollen state and device disintegration can be prevented by utilizing TNAs for drug release from implantable devices.^13–15^

Despite the extensive development of TNAs for orthopedic and dental applications, the use of TNAs as a multifunctional material in soft-tissue applications remains largely unestablished. Both hard and soft tissues exhibit mechanosensitivity, which presents important design considerations for implants. Mechanically matched scaffolds promote bone ingrowth in orthopedic implants, enabling a mechanically robust biotic–abiotic interface that can withstand applied loads.^16^ In contrast, soft tissues are characterized by elastic moduli orders of magnitude lower than bone and frequently undergo large deformations. Therefore, it is often advantageous to use implant materials and designs that permit similarly dynamic conformational changes.^17^ Additionally, *in vitro* studies on 2D gels have shown a substrate modulus dependence on the proliferation and characteristics of cells found in the brain, such as neurons, astrocytes, and oligodendrocytes.^18^ *In vivo* studies have demonstrated that high-modulus materials implanted in soft tissues evoke a more pronounced inflammatory response than soft polymeric materials.^19^ As soft-tissue interfaces are often instrumented with integrated electrodes for monitoring or modulating tissue and cellular activity, the implant materials and device architectures should be chosen to maintain intimate contact between the implanted device and the targeted tissues. Therefore, flexible implant materials are often chosen for soft-tissue-interfacing applications, and the performance of the implant depends on its ability to match the soft-tissue characteristics.^17^

Most reports on TNA fabrication focus on their growth on rigid titanium substrates using electrochemical anodization.^20–22^ Uniform nanotube arrays with adjustable pore size, tube length, and wall thickness can be achieved by tailoring the electrochemical anodization parameters such as the applied voltage, reaction time, anodization temperature, and electrolyte composition.^23^ Zhu et al. reported the transfer of anodized TiO_2_ nanotube layers onto indium tin oxide (ITO)-coated poly(ethylene terephthalate) (PET) substrates with the aid of TiO_2_ nanoparticle pastes.^24^ Very few studies have described the direct growth of TNAs on flexible substrates for various applications such as biosensors.^25–27^

This work aims to advance the use of TNAs in flexible implants for soft-tissue (e.g., nervous system)-interfacing applications by developing methods for integrating TNAs on polymer substrates. Here, we evaluate the cytotoxicity of TNAs in response to microglial cells and explore the drug-release behavior of TNAs-on-polyimide substrates in different bending modes. In addition, we demonstrate approaches for incorporating electrochemically anodized TNAs on two different polymer substrates using microfabrication techniques. As a result, a multifunctional platform has been developed, which combines the drug-releasing properties of biocompatible TNAs with the mechanical flexibility of the polymeric substrate. To the best of our knowledge, this report is the first demonstration of the fabrication of TiO_2_ nanotube arrays on flexible substrates for application in implantable biomedical devices.

## Results

### Evaluation of microglia *cytotoxicity* of TNAs

**Figure 1:**
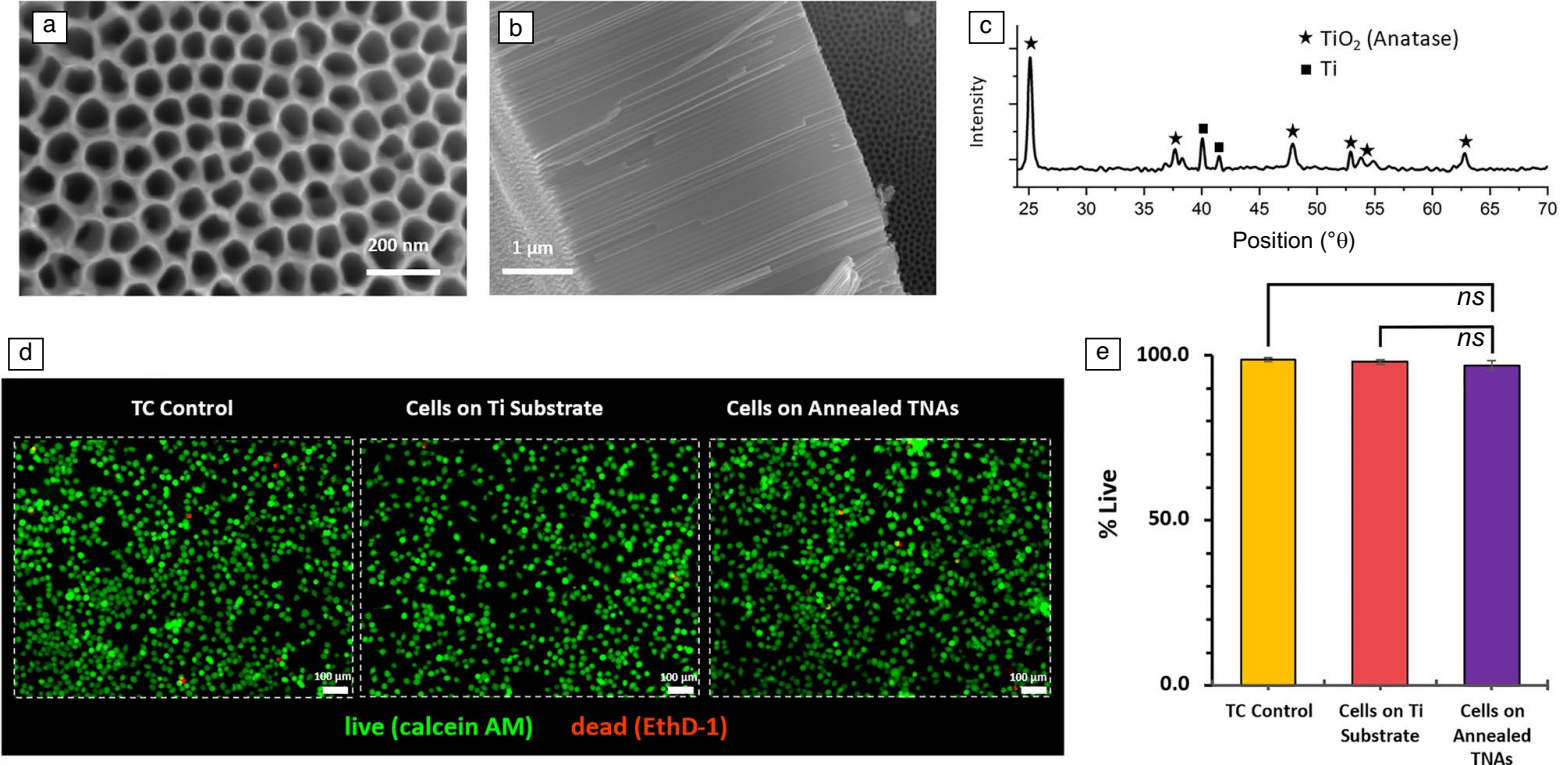
(a) Top and (b) cross-sectional view scanning electron microscopy images of nanotubes anodized at 40 V. The nanotubes’ pore diameter ranges from ~80 to 100 nm, and the nanotubes’ length is ~5 µm. (c) X-ray diffraction pattern recorded for an annealed titania nanotube array (TNA) sample in air at 450°C for 4 h, confirming the transformation of nanotubes to crystalline anatase phase. (d) Fluorescent images revealing the morphological features of microglial cells cultured on the well plate (left), on Ti substrate (middle), and on annealed TNA samples (right) after 48 h; (e) live/dead assay results highlighting the viability of highly aggressively proliferating immortalized microglial cells cultured on the bare well plate, Ti, and annealed TNAs for 48 h. Data are presented as mean ± SD (*n* = 4); *ns* no significance. Statistical significance was considered when *p* value <0.05. TC, tissue culture.

First, this study aims to examine the biological response of soft tissues to TNAs using brain-derived cells. Microglia cytotoxicity of TNAs was evaluated using TNAs grown on Ti foils. **Figure ****1**a–b illustrates the top surface morphology and cross-sectional view SEM images of highly ordered vertically aligned nanotube arrays after annealing, with pore diameter ranging from ~80 to 100 nm and a tube length of ~5 µm achieved by anodizing the Ti foil at 40 V, for 100 min. The XRD pattern of the annealed TNA sample in air at 450°C for 4 h confirmed the transformation of nanotubes to crystalline anatase phase (Figure 1c). The fluorescent images (Figure 1d) show that the annealed TNAs sustain the survival of the highly aggressively proliferating immortalized (HAPI) microglial cells, as demonstrated by green-stained live cells versus red-stained dead cells, with no significant differences in cell viability compared to TC (tissue culture) control and bare Ti substrate as evidenced by the live/dead assay and statistical analysis results shown in Figure 1e. Data are expressed as mean % of cell viability ± standard deviation (SD) for *n* = 4 samples per group.

### Direct growth of TNAs-on-polyimide substrates

**Figure 2:**
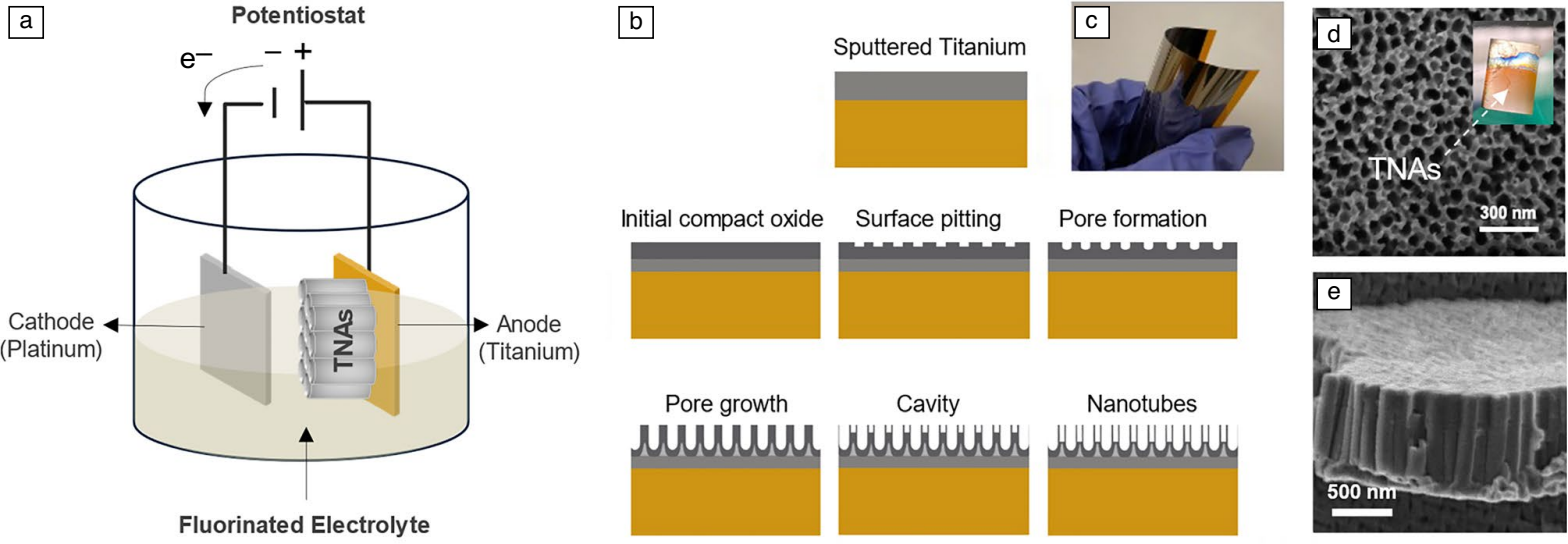
Schematic diagrams of (a) electrochemical anodization setup and (b) formation mechanism of titania nanotube arrays (TNAs) from Ti-sputtered polyimide substrate. (c) Photo of a flexed Ti-sputtered polyimide substrate. (d) Top-view and (e) cross-section scanning electron microscopy images of a freestanding piece of TNA grown on polyimide substrate. The inset in (d) shows the transparent as-anodized TNAs-on-polyimide substrate.

Schematic diagrams of the electrochemical anodization setup and formation mechanism of TNAs on sputter-deposited Ti on a polyimide substrate are shown in **Figure ****2**a–c. By anodizing the thinner Ti-sputtered polyimide samples (Ti thickness ~1 µm) at 40 V for 30 min, TNAs with an average pore size of ~85 nm and a tube length ~1 µm were achieved (Figure 2e–d). The wall thickness of nanotube arrays on the polyimide substrate is much thicker than tubes grown on the pure Ti substrate. This could be due to the tapered shape of the nanotubes, where the walls are thicker at the bottom and thinner at the surface, resulting in thicker walls after a shorter anodization time. Nanotube walls become thinner near the surface as they grow longer, resulting in larger pores near their surfaces.^28^ By monitoring the anodization current profile over time, the anodization of Ti-coated polyimide was stopped when the critical steps for anodic oxidation of titanium film was achieved. These critical steps include TiO_2_ formation by initial oxidation of the titanium layer, and then the nanoporous or nanotube formation, followed by electric-field-assisted and chemical dissolution of TiO_2_, as discussed previously.^29^ The formation of nanotubes from sputtered thin-film Ti (on non-Ti substrates) is very sensitive to the electrolyte composition, anodization time, and post-cleaning; without these considerations, the nanotube layer can easily be dissolved or damaged.^30^

### Release of the anti-inflammatory dexamethasone from TNAs-on-polyimide in different bending modes

**Figure 3:**
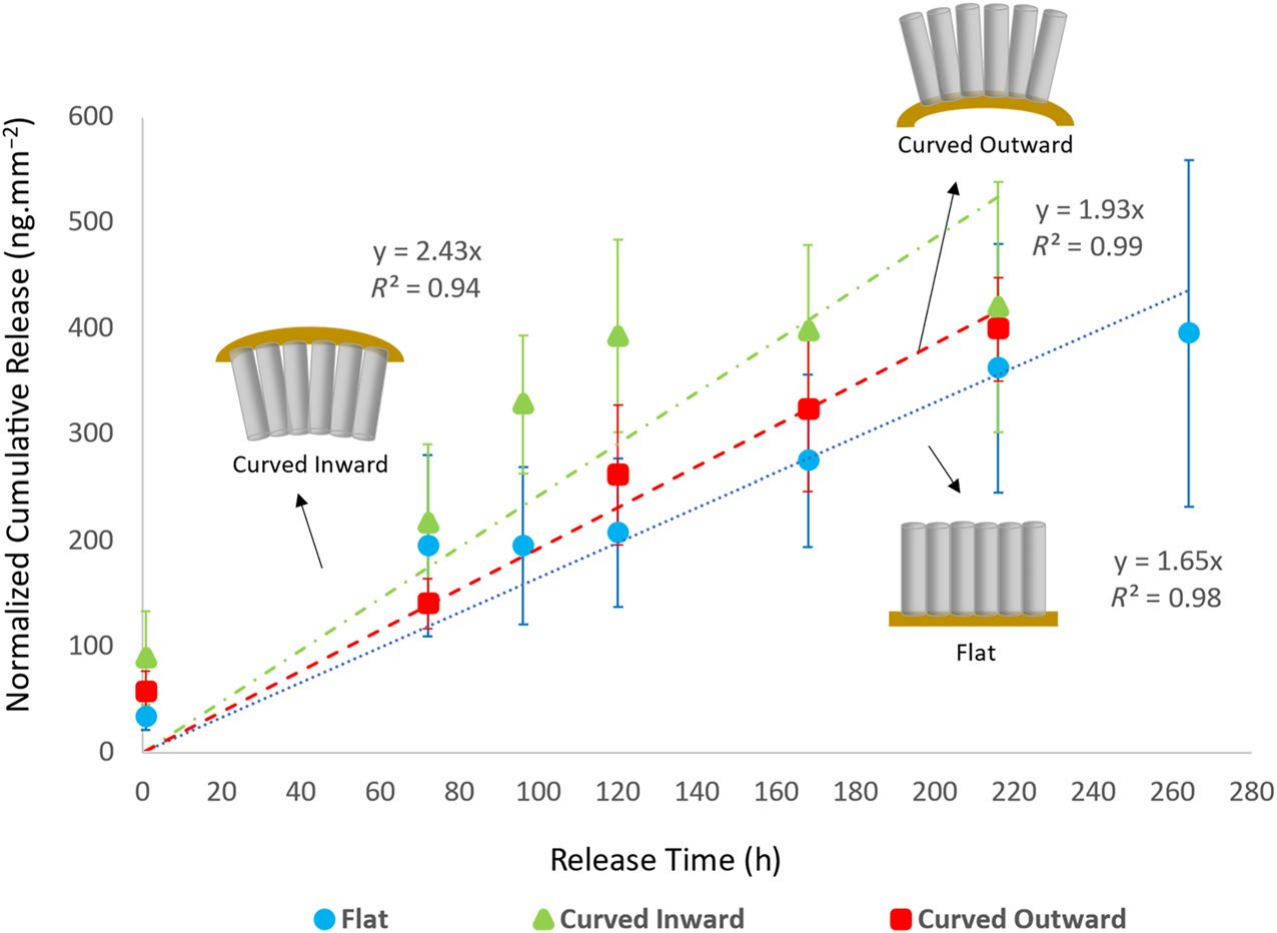
Comparison of *in vitro* cumulative DEX release rate profiles of titania nanotube arrays (TNAs)-on-polyimide samples in different bending modes at 37°C: flat, curved inward (*R*_c_ = 5 mm), and curved outward (*R*_c_ = 5 mm). The release data show the normalized cumulative release averaged over three samples in each group and obtained from TNAs-on-polyimide samples with ~1-µm-long nanotubes and ~85-nm-diameter pores, which goes on for ~10 days. Insets: Schematics of the samples (not to scale) in flat, curved inward, and curved outward bending modes. Error bars show standard errors of the mean (*n* = 3). Statistical significance was considered when *p* value <0.05.

The current study used dexamethasone (dexamethasone 21-phosphate disodium salt, hereafter named as DEX), a commonly used anti-inflammatory, as a model drug. Dexamethasone is considered a small molecule and a negatively charged ion with low molecular weight of 516.4 g mol^−1^. **Figure ****3** compares *in vitro* cumulative DEX release rate profiles of TNAs-on-polyimide samples at 37°C in three different bending modes: flat, curved inward, and curved outward (as illustrated in schematics). The release data are obtained from TNAs with ~1-µm-long tubes and 85-nm-diameter pores (*n* = 3 per group) as shown in the top and cross-sectional view SEM images of a freestanding piece of TNAs detached from the polyimide substrate for imaging purposes (Figure 2d–e). *In vitro* release rate studies on samples at different bending conditions were conducted to mimic the potential deformation of the nanotubes in flexible devices while implanted and to assess the effect of sample bending on drug-release behavior.

Prior to cutting the large coupon of TNAs-on-polyimide (area ~62 mm^2^), a 2 mL volume of 24 µM DEX solution (molecular weight of DEX = 516.4 g mol^−1^) was loaded on it (see Supplementary information, Figure S1a) resulting in loading 24.8 µg of DEX into the sample. Therefore, a total amount of 400 ng mm^−2^ is expected to be released from each cut sample. Figure 3 shows the release profiles of all three groups of samples (expressed as cumulative mass of DEX eluted, averaged over three samples within each group, and normalized to sample area). The first data points for all samples in all three groups were obtained at *t*_1_ = 45 min after the samples were placed in 1 X PBS. These initial nonzero release at the beginning of the process at *t*_1_ = 45 min is an indicative of fast diffusion of DEX molecules due to high-concentration gradients between TNAs’ interface and PBS solution. In addition, this could be related to dissolution of DEX that stayed on the surface of TNAs (not loaded inside the nanotubes). The release data are plotted up to the time point at which the samples stopped releasing to enable linear fitting; this time point corresponds to the time the total DEX amount released from each group reached ~400 ng mm^−2^. This is also approximately equivalent to 100% DEX released when normalized to the loaded mass per sample area. The release measurements for three groups were continued for as long as there was increase in cumulative release. Table S1 (in Supplementary information) summarizes the normalized cumulative DEX release values corresponding to the last measured time points for each group. The reductions in DEX cumulative release (negative release rates) for flat and curved inward groups were found to be within the error range. However, the curved outward group showed larger negative release outside the error range; this could possibly be related to partial movement of DEX molecules back into the nanotubes resulting from a large concentration gradient between the reservoir and the empty nanotubes in our closed setup. This effect is unlikely to occur *in vivo* because drugs are rapidly absorbed by surrounding tissue.

The release data indicate that when nanotubes are bent inward or outward, respectively (*R*_c_ = 5 mm, Figure S1b–c), the majority of loaded DEX is eluted within 5–9 days, while DEX release from the nanotubes in a flat condition can extend to 11 days (or 10 days, because no measurements were performed between day 9 and day 11 for this condition). Using ImageJ software, the available pore volume of the (~62 mm^2^) TNAs-on-polyimide sample with ~1-µm-long tubes was found to be ~50% of the nanotube film volume (Figure S1d–e). This volume can allow ~41 µg of DEX (density = 1.32 g cm^−3^) or 660 ng mm^−2^, which is ~40% more than the loaded amount. Therefore, by filling the nanotubes to their maximum volume, and with samples maintaining the same release rates, we can expect DEX release from TNAs for up to 20 days.

The release kinetics of DEX from TNAs-on-polyimide samples in curved inward condition showed the highest average release rates of ~2.43 ng mm^−2^ h^−1^ (as indicated by the slope of the fitted line) while the sample in curved outward and flat conditions showed average release rates of ~1.93 ng mm^−2^ h^−1^ and ~1.65 ng mm^−2^ h^−1^, respectively. The lower *R*^2^ value of ~0.94 for the curved inward condition (compared to those of ~0.99 and ~0.98 for curved outward and flat conditions) is indicative of the curved inward samples deviating from linear release kinetics due to a slightly rapid release of DEX by day 5 and a slowed release over the next 4 days, as shown by the data (triangles) in Figure 3. To validate the significance of *in vitro* release rate of DEX from TNAs at different bending conditions, a series of linear regressions were conducted using dummy variables to assess differences in slopes between different groups. A *p* value < 0.05 was considered statistically significant in all analyses. Results showed a significant difference in slopes (release rates) of the flat samples and samples flexing inward. However, no significant differences were observed in terms of the release rates between flat samples and samples flexing outward (and also between samples flexing inward and outward) at a radius of curvature, *R*_c_ = 5 mm (Table S2).

### Patterning of TNAs-on-polyimide substrates

Extending therapeutic release beyond the duration exhibited by ~1-µm-long TNAs grown from sputtered Ti on a polyimide substrate requires the longer nanotubes afforded by Ti foils, which can range in thickness from 10 µm to several millimeters. Additionally, the thinner walls for foil-derived TNAs enable a higher drug-loading efficiency than the thick-walled TNAs derived from sputtered Ti. Furthermore, annealing of TNAs grown from sputtered Ti on polyimide or other polymer substrates is limited to the glass-transition temperature or melting temperature of the polymer substrate. The melting temperature for the type of polyimide used in this study is ~400°C, which is sufficiently high for crystallizing the amorphous TNAs to anatase;^26,29^ however, high-temperature processes cannot be performed on most polymers. Thus, to facilitate integrating fully processed TNAs onto polymer substrates and to enable a high degree of mechanical flexibility, TNA microsegment islands were patterned from foil-derived TNAs.

**Figure 4:**
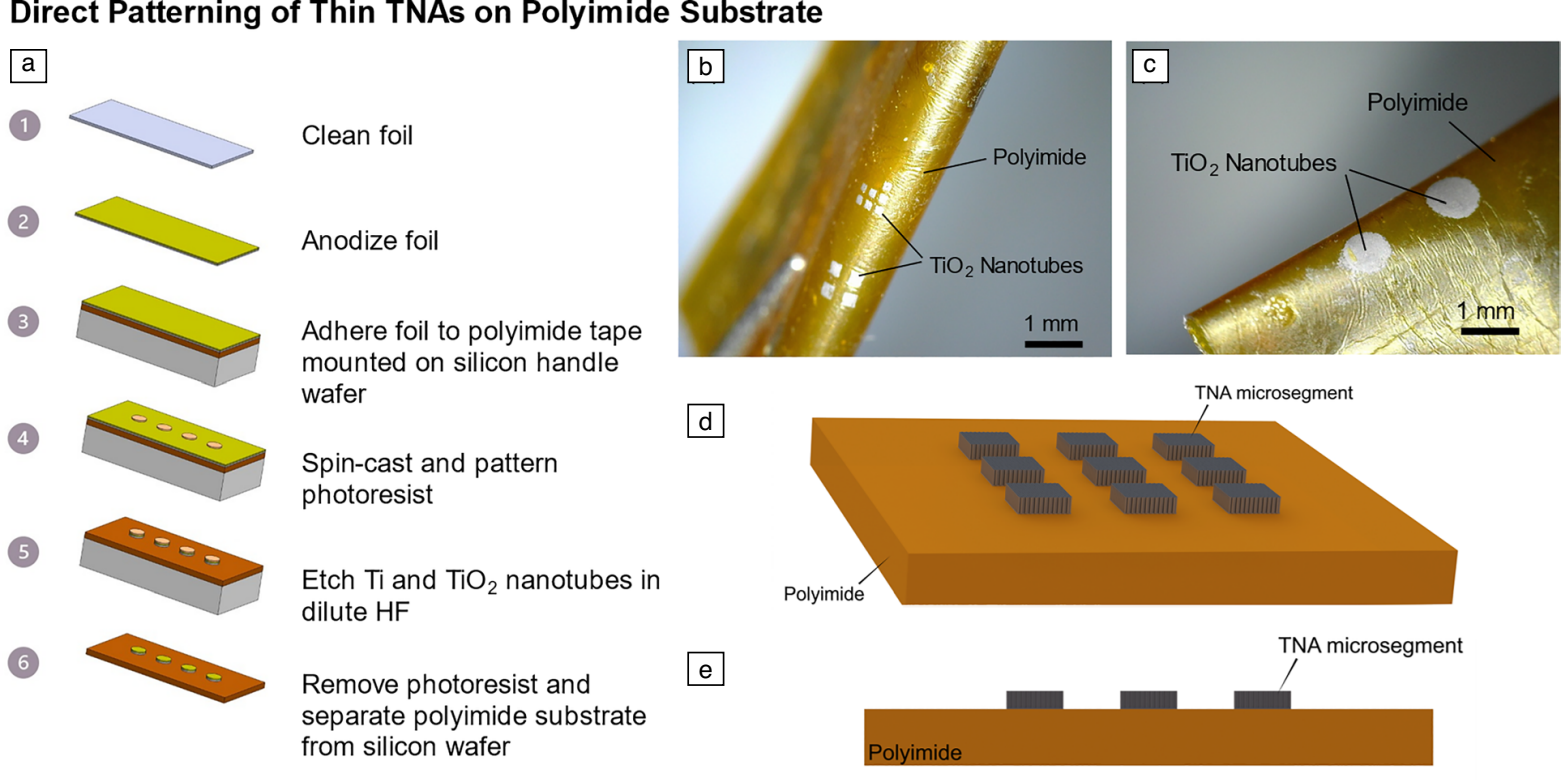
(a) Process flow for direct photolithographic patterning of titania nanotube arrays (TNAs) on a polyimide tape with a silicone-based adhesive. In this case, the TNAs were mounted TNA-side up onto the polymer, then wet-etched through a photoresist mask. (b) and (c) Raised TNA microsegments on the polyimide tape with a range of feature sizes. The tape is flexed with a small curvature (*R*_c_ < 1 mm), demonstrating the flexibility of the devices with the rigid islands. (d) Three-dimensional and (e) cross-sectional view schematics of patterned TNA microsegments on the polyimide substrate. Only the bottom of the Ti/TNA foil interfaces with the substrate via a silicone adhesive layer.

The first method for forming microscale TNA segments involved photolithographically patterning a 10-µm-thick Ti/TNA foil adhered to a 25-µm-thick polyimide-based tape with a silicone-based adhesive layer (**Figure ****4**a). The TNAs were selectively masked using photoresist, and the unprotected areas were removed using an HF-based etchant. For as-anodized TNAs on the Ti foil, the HF-based wet etchant etched both the TNAs and the underlying Ti. After photoresist removal with acetone, raised TNA islands on top of the polyimide tape formed a pattern corresponding to the photoresist mask (Figure 4b–e). Upon flexing the polyimide foil, the TNA microsegments remain intact with no visible delamination, cracking, or deformation. The success of this direct-patterning method is highly dependent upon making conformal contact between the TNA foils and the adhesive on the polyimide-based tape. Any air gaps between the foil and the adhesive will lead to the removal of the TNA microsegments during the wet-etching process. This fabrication process is compatible with low-temperature substrates, with all processes taking place at room temperature apart from a brief 60-s exposure to 110°C on a hot plate for photoresist processing. Therefore, this process can be applied to various substrate materials when accompanied by an adhesive layer.

### Patterning and transferring TNA films to polymer substrates

**Figure 5:**
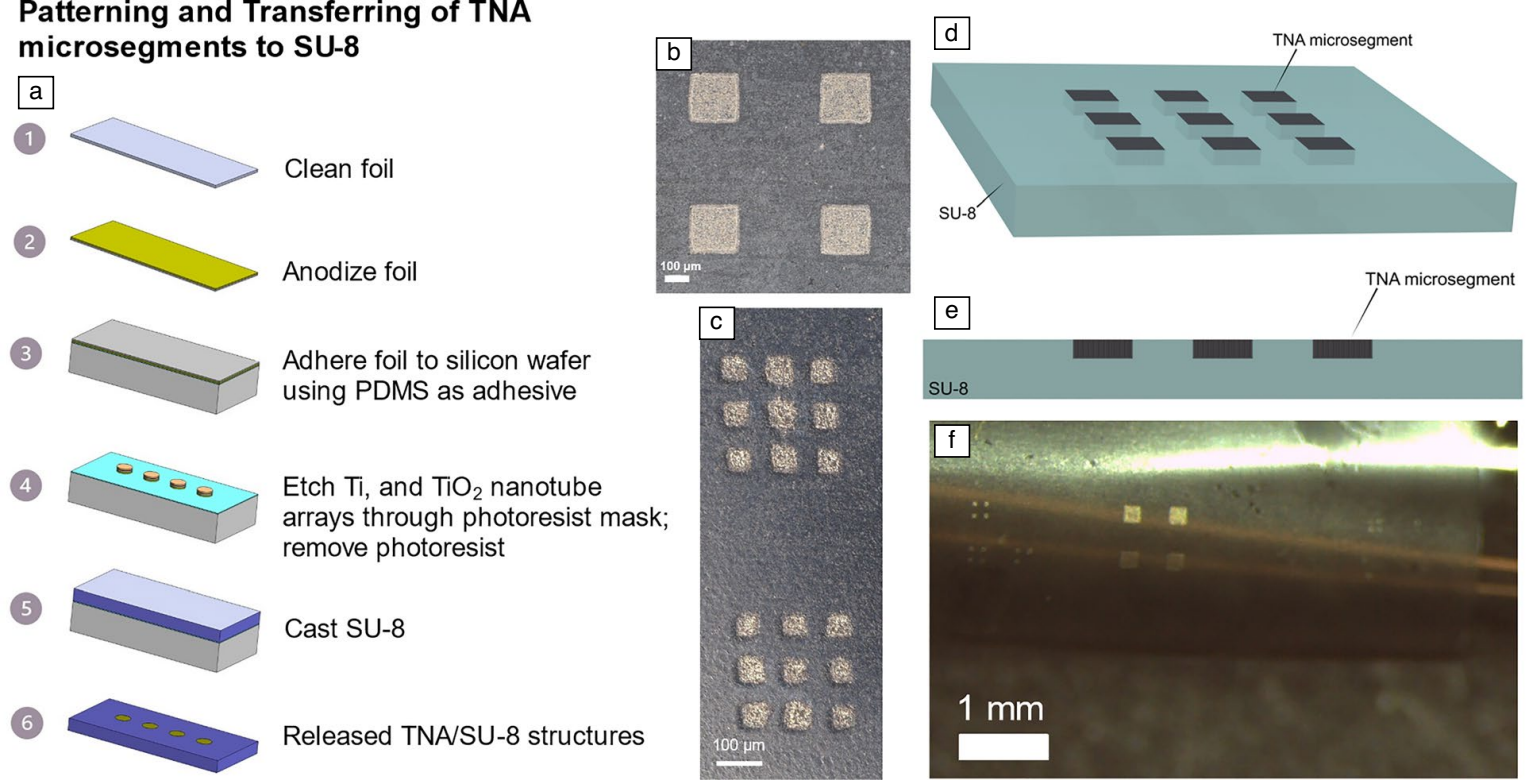
(a) Process flow for patterning titiania nanotbe arrays (TNAs) on a PDMS-coated silicon wafer, then transferring to an SU-8 film. In this case, the TNAs were mounted TNA-side down on the PDMS, then wet-etched through a photoresist mask. SU-8 was then spin-cast on top of the wafer and cross-linked. When the SU-8 was separated from the PDMS, the TNA microsegments preferentially adhered to the SU-8 and were released from the PDMS. (b, c) TNA embedded within SU-8, with only the top surface of the TNAs exposed. (d) Three-dimensional and (e) cross-sectional view schematics of patterned TNA microsegments on the SU-8 substrate. The bottom and the sidewalls of the TNA microsegments interface with the SU-8. (f) The TNAs remain securely in place, even when flexing the SU-8 substrate (*R*_c_ ~1 mm), demonstrating the flexibility of the devices with the rigid islands.

To reduce mechanical stresses on the TNAs during flexing and to further improve the mechanical robustness of the interface between the TNAs and the polymer substrate, a transfer technique was developed. In this case, the TNA foils were patterned TNA-side down while adhered to a poly(dimethylsiloxane) (PDMS)-coated silicon wafer (**Figure ****5**a). The TNA adhesion to the PDMS layer was sufficiently strong to facilitate photolithographic patterning of microsegments as small as 20 µm × 20 µm. Given the use of an isotropic wet etchant, it was important to thin the 25-µm-thick foils to 7 µm by etching most of the Ti prior to photoresist application. After patterning the TNA microsegments, a 20-µm-thick SU-8 was spin-cast and cross-linked on top of the TNAs. This coated the exposed backside surface of the TNA foils, as well as the exposed TNA microsegment sidewalls with SU-8, while the top side of the TNAs was sealed against the PDMS. Upon peeling the SU-8 from the PDMS-coated wafer, the TNA microsegments were transferred to the SU-8 film. This resulted in TNA microsegments embedded within the SU-8, with only the top surface of the TNA microsegments exposed to the environment (Figure 5b–e).

PDMS was chosen as the adhesive for this process because it provides sufficient adhesion to the TNAs to hold the microsegments precisely in position during the patterning process, but the TNAs then preferentially adhere to the SU-8 when separated from the PDMS. Integrating TNAs into a SU-8 substrate does not require an adhesive, as the SU-8 has relatively strong adhesion to Ti, largely due to electrostatic interactions.^31^ This technique can be extended to integrate TNA microsegments into various solution-castable or vapor-deposited polymers by relying upon mechanical interlocking and van der Waals interactions to hold the microsegments in place.

## Discussion and conclusion

The first objective of this work was to evaluate the impact of the annealed TNA surface on microglial cell viability via *in vitro* study. Microglia are the primary immune cells of the central nervous system (CNS) and account for 10–15% of all cells found within the brain. Microglia play an important role in maintaining the health of the CNS by the removal of pathogens, infectious agents, and damaged cells through phagocytosis.^32^ Microglia have also been extensively studied for their roles in neuroinflammatory diseases and neuroinflammation following intracortical microelectrode implantation.^33,34^ The highly aggressively proliferating immortalized (HAPI) mouse microglial cell line is one of two microglial cell lines that are not genetically modified and are derived from an enriched postnatal day 3 mouse primary microglial culture.^35^ As seen in Figure 1d, after 48 h incubation—which is an adequate time to reveal the acute inflammatory response to the surface of the implant–cell retention and survival (percent live cells) on the annealed TNAs was similar to untreated controls. Our findings were found to be consistent with previous studies demonstrating that annealed TiO_2_ nanotube arrays do not produce cytotoxic results on hard connective tissues.^36–38^ However, interfacing TNAs with soft-tissue/organ systems requires similarly soft device structures to minimize tissue damage and facilitate tissue integration.^39–41^

The formation of TNA on metal or polymer substrates is largely affected by the grain structure of the Ti layer as well as the charge transfer properties of the substrate in contact with Ti.^25^ Unlike pure metal substrates, anodizing metals on low-conductivity substrates is more complex. Previous studies have reported that the deposition conditions, such as substrate temperature, can change the quality of the sputtered film and directly impact the formation of highly ordered TNAs.^25^ Among polymeric substrates, polyimides are high-performance polymers that are considered promising materials for biomedical implants due to their nontoxicity and excellent long-term *in vivo* stability.^42,43^ Polyimides such as Kapton HN are popular for their high thermal conductivity and relatively high resistance to high temperatures (up to 400°C). As such, they are an excellent choice for the deposition of high-quality titanium films, which is required for the formation of highly ordered titania nanotube arrays. A further advantage of the Kapton HN substrates is that they can withstand the high-temperature annealing process, thus, allowing for the formation of crystalline (anatase) TNAs.^26^

An important characteristic of the anodization process is its controllability and versatility to produce TNAs with desired dimensions (e.g., pore size and length of nanotubes) by varying anodization potential and time, as demonstrated in our results in Figure S2. No structural changes were observed for as-anodized nanotubes soaked in PBS at 37°C over six months (Figure S3). This suggests TNAs will maintain their structural integrity for long-term *in vivo* applications, even without further processing. The annealing process resulted in the transformation of amorphous as-anodized TNAs to crystalline anatase (Figure 1c). Previous studies have shown that TNAs become more robust and their attachment to titanium substrates is enhanced after annealing due to the crystallization of TNAs and elimination of fluoride (F^−^) during the high-temperature annealing process, providing enhanced robustness to physiological conditions.^44,45^ In addition, cell viability exceeded 95% (*p* < 0.05) when microglial cells were cultured on the surfaces of annealed TNAs with pore diameters of ~80–100 nm (Figure 1d). According to other studies, the nanotopography of the microenvironment is a dominant factor influencing the cell interactions, whereas the crystalline structure or fluoride content of TiO_2_ nanotubes has less impact.^37^ For other types of tissue, such as epithelial and connective tissue, the viability of endothelial cells and bone marrow mesenchymal cells (MSCs) on TiO_2_ nanotube surfaces was shown to be dependent on the pore size—with higher activity reported for 15–70-nm pores—but independent of the nanotube’s fluoride content and crystalline structure.^36,37^ Thus, it is expected that microglial cells also perform better on pores smaller than 80 nm compared to other types of tissue and respond similarly when in contact with as-anodized TNAs (which contain fluoride in their composition) for TNAs grown on substrates that cannot withstand the high-temperature annealing.

A major difference between the diffusion-driven drug-release mechanism in TNAs and the polymer-based materials is that the surface chemistry and geometric properties of the TNAs, the relative size of drug molecules and nanotube pore, and the organization of the drug molecules in the nanotubes can all be utilized to tailor linear release kinetics relevant to different therapeutic requirements.^9,13^ As a result, this material system offers an alternative to polymer-based drug-release systems that typically show burst releases with potential risks of reaching toxic levels, followed by rapid declines in release rate, resulting in complete depletion within hours or days.^14,46^

In addition to controlled release characteristics, nanotube morphology can be tuned via anodization to optimize release profile for various types of drugs. Multiple factors contribute to the loading and release of drug molecules in TNAs. In order to determine which drugs are suitable for use in TNAs system, several physical and chemical characteristics of the drug must be examined with respect to TNAs. The main characteristics include molecular weight (which represents the size for most of the molecules), solubility, and electrostatic charge of the drug. Low molecular weight (or namely small) drug molecules within the range of Da to kDa can be considered for application in TNAs with ~100-nm-diameter tubes.^47^ In general, the drug-loading efficiency can be influenced by the type of solvent used for loading as well as the solubility limit of the drug molecule, the concentration of the loading solution, and the number of micropipetting used to fill the drug molecules within the nanotubes. In particular, the release of drug molecules from TNAs can be regulated by interactions between negatively or positively charged molecules and the slightly negatively charged hydrophilic surface of titania nanotubes due to the presence of terminal hydroxyl groups, which could lead to faster or slower release rates.^48^

In terms of *in vivo* applications, previous studies reported that DEX can modulate the inflammatory response to neural implants at concentrations exceeding 0.2 µM and can cause neurotoxic effects at 100 µM, providing a relatively wide therapeutic window that can be easily targeted by TNA designs.^49,50^ While the statistical analysis of release data revealed a significant difference between the curved inward and flat groups, the release rates obtained from flexible TNAs at different bending modes indicated that flexible TNAs can maintain the local DEX concentration within the targeted range for efficacy for the entire release duration without exceeding the threshold for toxicity; therefore, flexible TNAs can be utilized as a controlled drug-release platform for interfacing with implantable devices for a wide range of applications.

Noting the potential for TNAs to provide drug-release functionality for many biomedical implant applications, we sought to develop a set of fabrication methods that would enable TNA integration with materials that are already used for interfacing to soft tissues.^51–55^ One approach is to deposit a uniform layer of titanium on the polymer substrate, followed by electrochemical anodization. Sputtering techniques can produce high-quality films with good adhesion to the substrate. However, a maximum film thickness of ~2-µm-thick films limits the tube length and, therefore, are suitable only for short-term drug delivery applications.

Sustained delivery applications require longer nanotubes (e.g., >2 µm).^56^ According to previous studies, release of drug could be prolonged by varying other parameters such as loading the nanotubes with higher amounts of drug or lowering the release temperature.^57^ Increasing the length of TNAs can become problematic when applied on a flexible substrate. Due to their brittle nature, TNAs could fracture when subjected to intrinsic or applied stresses. When a thin film is deposited to a flexible substrate, it will induce stress to the substrate, which will result in bending the substrate. This stress can change during the anodization process due to volume expansion resulting from metal to oxide conversion as well as voltage-induced electrostrictive forces.^58^ If TNAs are grown on one side of the substrate, the substrate bends with a curvature $1/{R}_{\mathrm{C}}\propto {\sigma }_\mathrm{f}{t}_\mathrm{f}$, where ${\sigma }_\mathrm{f}$ is the average stress in the film and ${t}_\mathrm{f}$ is the thickness of the film.^59^ Intrinsic stresses (compressive or tensile) become more significant with increasing TNA thickness. This will be of particular concern when flexible substrates with low Young’s moduli (2–5 GPa) are used to mimic soft-tissue mechanics.^60^ To minimize stresses caused by increased sputtered Ti thickness, longer TNAs (or thicker films) on Ti foil can be used. However, the brittleness of the TNAs’ layer prevents the substrate from being flexed. To avoid this, longer TNAs on Ti sheet were patterned into microscale features integrated onto a flexible substrate to provide both the benefits of the nanotubes’ length and the benefits of flexible substrates. We used both direct-patterning and pattern-and-transfer approaches to integrate foil-derived TNA microsegments onto polymer substrates.

Photolithographic patterning was used to pattern foil-derived TNA microsegments either directly on the polymer substrate or on a temporary substrate before transferring to a polymer substrate. The polymer regions between the TNA microsegment islands relieve strain while the structure withstands mechanical manipulation. The rigid island strategy has been used by other groups integrating segments of rigid materials (e.g., active electronics) onto flexible and stretchable substrates.^61,62^ Direct patterning can be used when there is a means of adhering the TNA foil to the substrate and results in the formation of TNA microsegments on top of the substrate surface (Figure 4d–e). This approach is applicable to thin-film-derived TNAs as well and allows for complex device designs with few process steps. In the second approach, microsegments of TNA were encapsulated in polymer from both the bottom and sidewalls as part of a pattern-and-transfer process to ensure a well-integrated, mechanically stable structure for long-term implant applications (Figure 5d–e). The most significant advantage of this method is compatibility with high-temperature TNA post-processes that occur before integration with temperature-sensitive substrates. Therefore, these processes are not restricted to the well-established polyimide and SU-8 substrate materials. The pattern-and-transfer technique is highly versatile and can be adapted to various soft materials.

In summary, this work aimed to demonstrate methods for integration and the functionality of titania nanotube arrays on flexible substrates as a biocompatible drug delivery platform for soft-tissue interface applications. The viability of microglial cells on the surface of annealed TNAs with pores between 80 and 100 nm exceeded 95% (*p* < 0.05). Sustained linear release of anti-inflammatory dexamethasone was obtained from TNAs-on-polyimide substrates over a period of ~10 days; no distinct differences were observed in the release rates for samples under different bending modes. Direct growth of large-area TNAs from both thin-film Ti and Ti foil was successfully demonstrated. Further, direct-patterning and pattern-and-transfer methods were developed to integrate microscale TNA segments onto polymer substrates. The later approach allows for spatially defined integration of long TNAs while maintaining the mechanical flexibility of the polymer.

## Materials and methods

### Titanium deposition on polyimide

Titanium films (~0.3–1 μm) were deposited on Dupont 300HN polyimide (2-mil thick) substrates using a DC magnetron sputtering technique (Denton vacuum discovery 18, USA). A polyimide sheet was cut into 4 cm × 2 cm size pieces, and the pieces were ultrasonically cleaned with de-ionized (DI) water, ethanol, and isopropyl alcohol (IPA) and dried using a nitrogen gun. The polyimide pieces were then loaded into the sputtering chamber (Denton vacuum discovery 18). The base pressure was 8.4 × 10^–7^ Torr, and the power was 250 W. The film deposition process was conducted at 3-mTorr argon pressure, with a 75-mm distance between the cathode and substrate.

### Fabrication and characterization of TNAs

The electrochemical anodization of Ti foils and sputtered Ti on polyimide substrates was performed in a mixture of 0.5 wt% of ammonium fluoride (NH_4_F), 3 vol% of DI water, and 96 vol% of ethylene glycol (EG). For Ti foil anodization, high-purity-polished titanium foils (10-µm-thick, 99.99%, and 25-µm-thick, 99.98%, from Sigma-Aldrich and Futt, respectively) were sonicated in de-ionized (DI) water, ethanol, and isopropyl alcohol (IPA), separately, and then dried in air. The anodization was performed in a two-electrode setup with Ti as the anode and the platinum foil (99.99%, Sigma-Aldrich) as the cathode at 40 V for 30 min. The as-anodized samples on Ti foil were annealed at 450°C, for 4 h in air using a Lindberg/Blue MTM Mini-Mite Tube Furnace to convert the amorphous TiO_2_ nanotubes to crystalline anatase. The surface morphology of the films was observed using an Apreo 2 scanning electron microscope. X-ray diffraction was conducted using an x-ray diffractometer (Bruker AXS GmbH, D8 discover µMR, Cu*Kα*) for identification of crystalline phases.

### Cell culture

Highly aggressively proliferating immortalized (HAPI) cells were cultured in Dulbecco’s modified eagle medium (DMEM) supplemented with 10%FBS and 1%PS. TNA samples were autoclaved at 121°C for 10 min and were placed in 48-well plates using sterile forceps. A total of 5000 cells were seeded into each well of a 48-well plate containing one TNA sample in each well, and 1 mL of complete media was added on top of the nanotubes and cultured for 48 h. Live/dead cell viability/cytotoxicity kit (Invitrogen, Thermo Fisher Scientific) was used on cells after 48 h in culture. Cells were washed with Dulbecco’s phosphate-buffered saline (DPBS) and then stained for 30 min at RT with 25 nM calcein-AM solution and 100 nM ethidium homodimer-1 solution. The cell counts for live/dead discrimination were determined using Zeiss Axio Observer Z1 and ImageJ. Statistical analysis was performed using one-way analysis of variance (ANOVA), followed by post hoc Tukey comparison test at a 95% confidence level. All the data presented are expressed as mean ± standard deviation (SD) (*n* = 4). Statistical significance was considered when *p* value <0.05.

### Drug loading and release rate measurements

Drug loading on samples was performed by micropipetting a 24-µM dexamethasone solution (dexamethasone 21-phosphate disodium salt, Sigma-Aldrich, dissolved in 1:1 ethanol and DI water) onto the surface of a large coupon of TNAs-on-polyimide (area ~62 mm^2^). A total of 2 mL of DEX solution was pipetted onto the nanotube surface via several micropipetting steps. During these steps, the solution did not spill over the surface. After each application, the solution penetrated the nanotubes, and the solvent was allowed to evaporate for 1 h ensuring that the nanotube array surface was completely dry prior to each pipetting and the release studies. Then, the large coupon was cut into nine samples (flat, curved inward, and curved outward, three each). DEX-loaded samples were mounted on flat, curved inward (*R*_c_ = 5 mm), and curved outward (*R*_c_ = 5 mm) jigs and then immersed in Eppendorf tubes containing 300 µL of PBS (1× phosphate-buffered saline) and were tightly closed to avoid liquid evaporation. The tubes were placed in a water bath kept at 37°C. The released DEX concentration was obtained via absorption measurements on three 2.5-µL drops from each sample tube using a nanodrop one microvolume UV–Vis spectrophotometer at 242 nm. After the measurement, fresh PBS with the sampled volumes was added to the tubes. Daily measurements were taken for up to 14 days. A standard (calibration) curve with known concentrations of DEX was used to determine the unknown concentrations of DEX in the released medium.

### Statistical analysis of release rate data

To examine the statistical significance of the disparities between the fitted lines across distinct groups, specifically the “flat,” “curved inward,” and “curved outward” categories, a series of linear regressions were conducted using dummy variables to assess variations in slopes between different groups and to derive the corresponding *p* values. In all analyses, *p* values < 0.05 were considered statistically significant.

### Integration of TNAs with polymer substrates

TNA microsegments on the polyimide tape were patterned by wet etching through an AZ nLOF 2070 photoresist mask spin-cast on top of the tape. The foil was then etched in a 1% HF solution for approximately 10 min to remove all titanium and TNA between the defined microsegments. Upon etch completion, the photoresist mask was removed by rinsing with acetone and isopropanol. The process to pattern and transfer the TNA microsegments to a polymer substrate started with poly(dimethylsiloxane) (PDMS, Sylgard 184, Dow Corning) mixed in a 10:1 ratio, then degassed, and spin-cast to a thickness of 50 µm on a silicon wafer. The PDMS was partially cured for 15 min at 70^ο^C. Anodized (40 V/1.5 h) TNA/Ti foils were then placed TNA-side down onto the partially cured, tacky PDMS. Subsequently, the wafer was placed in an oven (1 h, 70°C) to fully cure the PDMS. At this point, the TNA/Ti foils were securely attached to the PDMS to facilitate further processing. Next, the TNA/Ti foils were thinned to approximately 6 µm by etching the Ti in 5% HF for 5 min. Using an AZ nLOF 2070 photoresist etch mask, the foil was then etched in a 1% HF solution for approximately 10 min to remove all titanium and nanotubes between the defined microsegments. After rinsing and drying, the photoresist was removed using acetone and isopropanol. At this point, patterned TNA microsegments were adhered to the PDMS coating the silicon wafer. To demonstrate the transfer process, SU-8 (SU-8 2015) was spin-cast onto the wafer to a thickness of 20 µm. The SU-8 was soft-baked, flood exposed with UV light, then post-exposure-baked. The SU-8 was then peeled from the PDMS, with the TNA microsegments transferring to and becoming integrated with the SU-8 film.

### Supplementary information

Below is the link to the electronic supplementary material.

Supplementary file1 (DOCX 18506 kb)

## Data Availability

The data are available from the corresponding author upon reasonable request.
