# Supplementary material for: Flexible multifunctional titania nanotube array platform for biological interfacing
Source: MRS Bull. 2023 Dec 8;49(4):299–309. doi: 10.1557/s43577-023-00628-y (PMC11026245; doi:10.1557/s43577-023-00628-y)
Supplement: Supplementary file 1 — Supplementary file1 (DOCX 18506 kb) [file 43577_2023_628_MOESM1_ESM.docx]

SUPPLEMENTARY INFORMATION

**
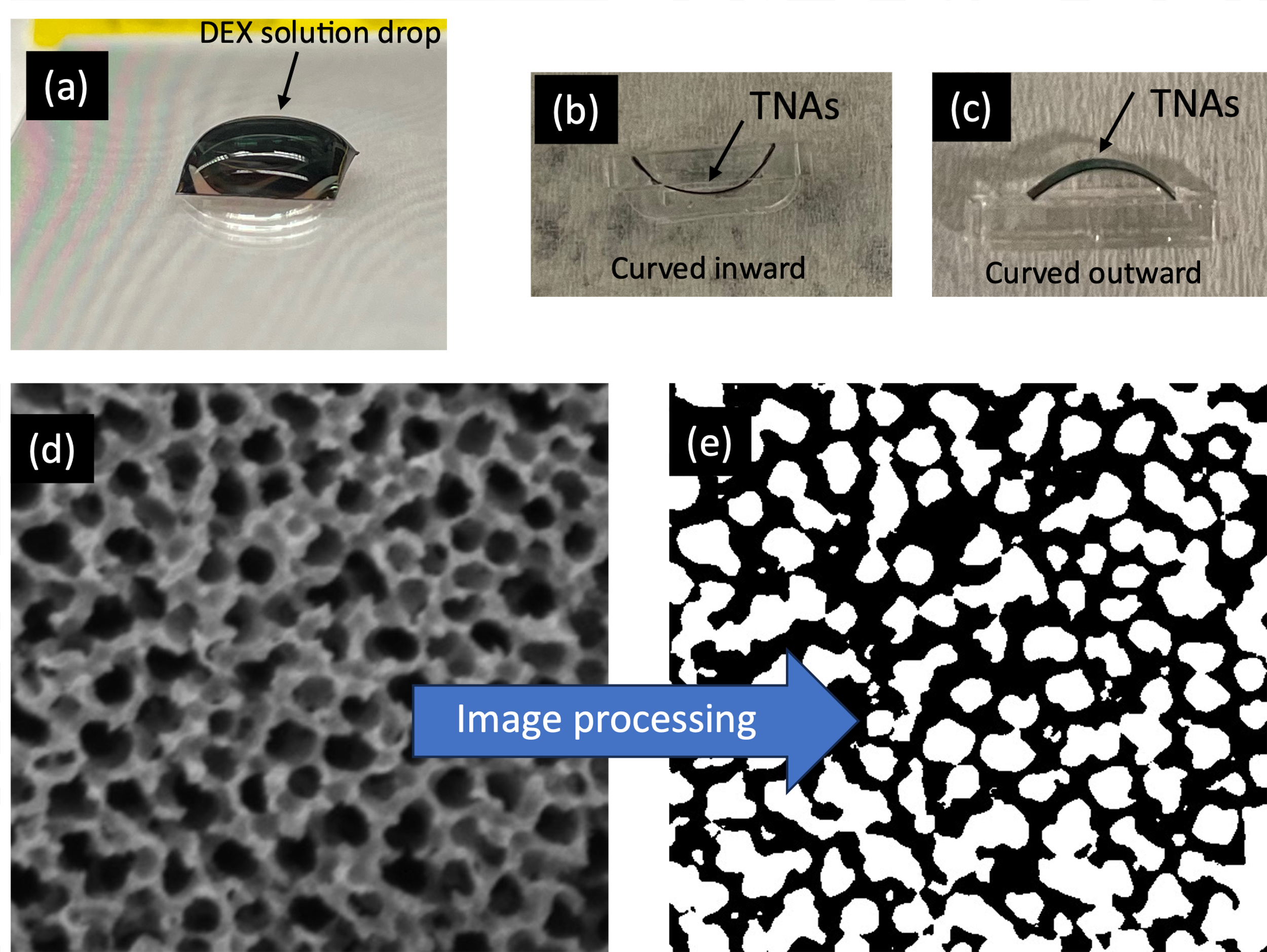
**

**Figure S1.** (a) photo of the TNAs-on-polyimide sample loaded with a drop of DEX solution. TNAs-on-polyimide sample mounted in jigs curved (b) inward and (c) outward. (d) SEM top-view image of TNAs grown on polyimide and (e) binary image obtained using ImageJ software for pore volume estimation.

**Table S1:** Normalized cumulative DEX release values corresponding to the last measured time points for each group.

**
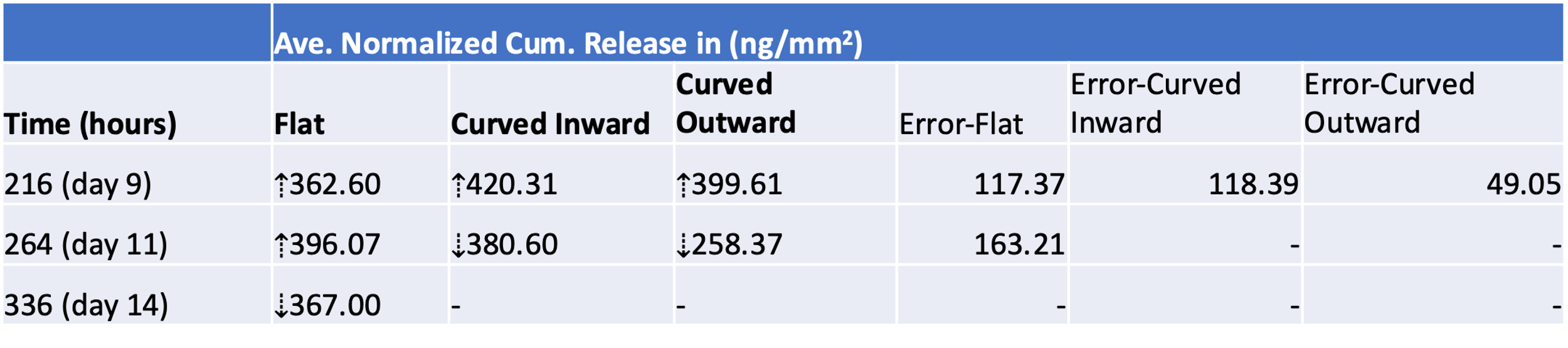
**

# Table S2: Statistical analysis (regression results) of drug release data on TNAs-on-polyimide samples in flat, curved inward and curved outward conditions.


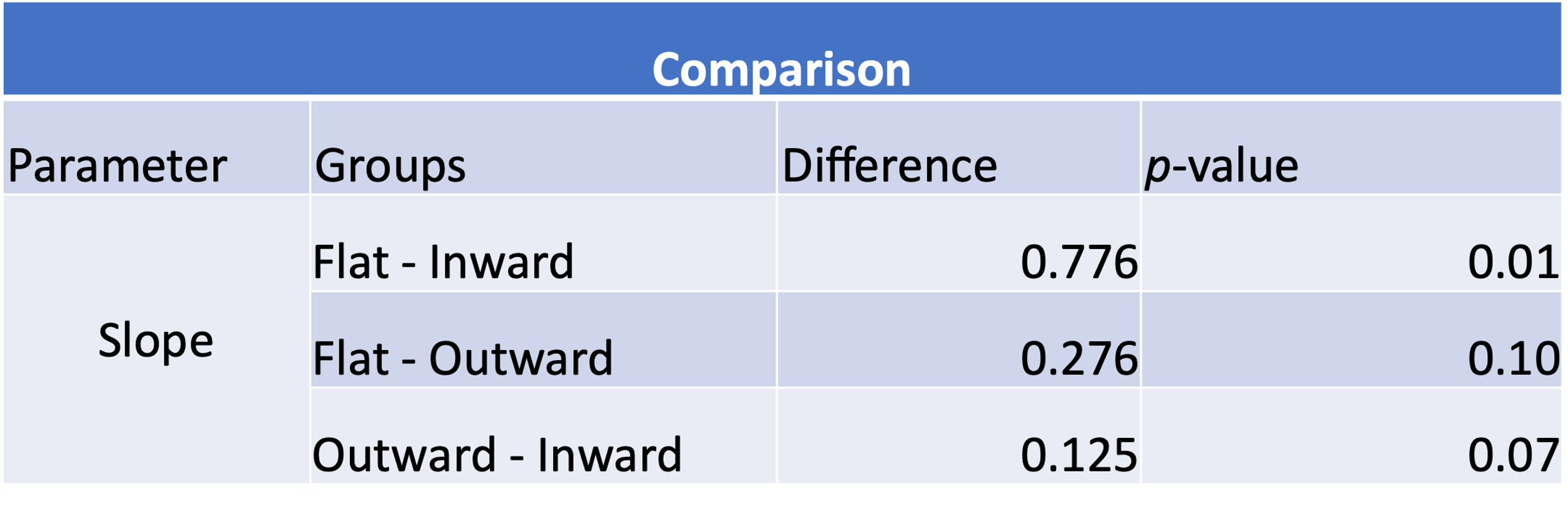


**
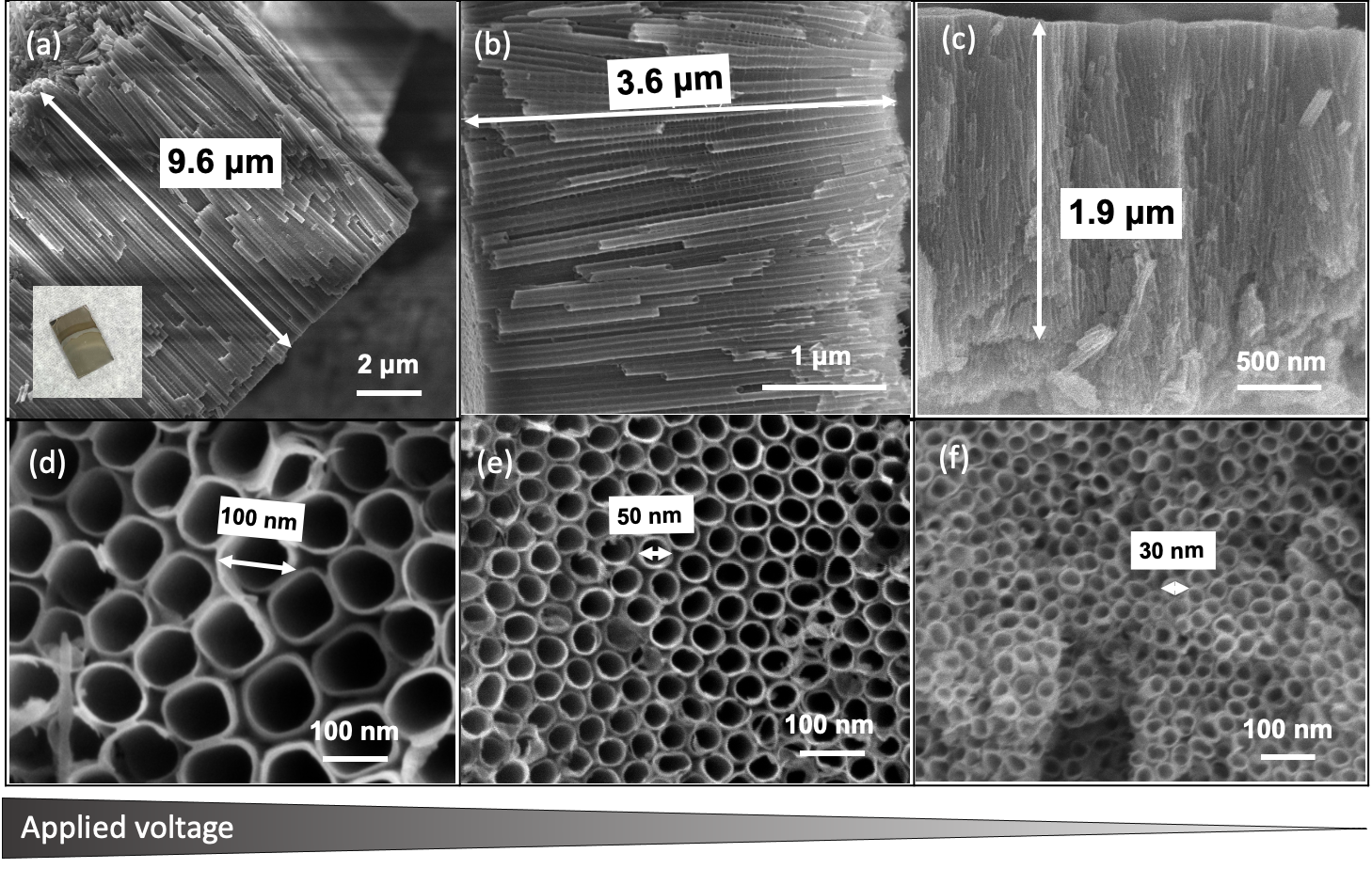
**

**Figure S2:** SEM cross-section (top row) and top-view (bottom row) images of TNAs grown with various tube lengths and pore sizes using applied potentials of (a, d) 40 V,
(b, e) 20 V and (c, f) 10V. The inset shows a photograph of a representative sample of size 8 x 5 mm^2^.


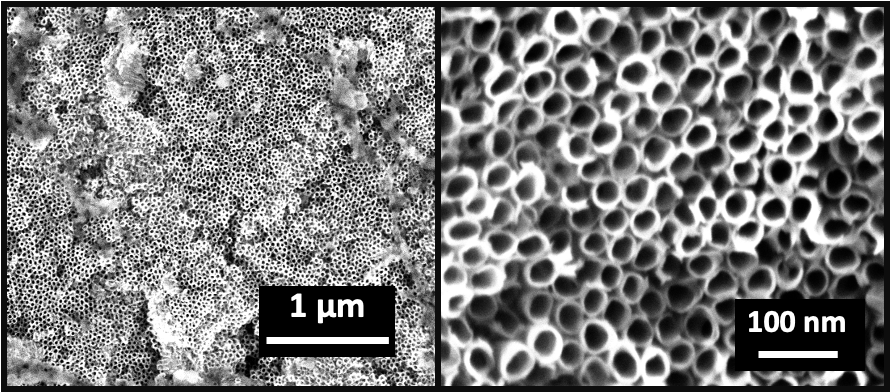


**Figure S3:** Top-view SEM images of the as-anodized TNAs after being soaked in PBS at 37°C for 6 months, indicating nanotube stability over a long duration at physiological conditions.
